# Eosinophilic Enteritis Flare-Up Mimicking Acute Gastroenteritis: A Rare Case

**DOI:** 10.7759/cureus.44199

**Published:** 2023-08-27

**Authors:** Maha Veer, Sapna Devi, FNU Sonia, Raja Ram Khenhrani, Mukesh Kumar

**Affiliations:** 1 Medicine, Liaquat University of Medical and Health Sciences, Jamshoro, PAK; 2 Internal Medicine, Shaheed Mohtarma Benazir Bhutto Medical College, Karachi, PAK; 3 Medicine, Azad Jammu Kashmir Medical College, Muzaffarabad, PAK

**Keywords:** diarrhea, vomiting, prednisolone, montelukast, dexamethasone, enteritis, eosinophilic gastrointestinal disorders, emergency

## Abstract

Eosinophilic enteritis is a rare subset of eosinophilic gastrointestinal disorders. It typically presents with chronic symptoms of abdominal pain, nausea, vomiting, diarrhea, and ascites. However, the clinical presentation can vary due to acute flare-ups. Here, we present a case of eosinophilic enteritis in a young female patient with intractable vomiting and diarrhea, mimicking acute gastroenteritis in the absence of other gastrointestinal symptoms. This case illustrates the challenge of diagnosing acute and diverse presentations of eosinophilic enteritis. It also highlights the importance of promptly treating and confirming the diagnosis through urgent tissue histopathology in adolescents with unexplained vomiting and diarrhea.

## Introduction

Eosinophilic gastrointestinal (GI) disorders constitute a heterogeneous group of rare diseases, including eosinophilic esophagitis, gastritis, enteritis, gastroenteritis, and colitis [1]. In 1937, Kaijser first defined the term “eosinophilic enteritis” (EE) by its association with GI symptoms and eosinophil-rich infiltration of the intestinal mucosa, without secondary intestinal eosinophilia [2]. After eosinophilic esophagitis, both eosinophilic gastroenteritis and EE represent the most frequent subset of disorders [3]. Although the disease has been described in case reports and reviewed multiple times, estimating its true incidence remains challenging due to many undiagnosed and unreported patients.

The exact pathogenesis of EE is unknown; however, the literature suggests the roles of immunoglobulin E (IgE)-mediated allergy and delayed T-helper type-2 (Th2) responses [4]. In most of cases, EE presents with non-specific and overlapping symptoms of abdominal pain, diarrhea, bloating, and nausea. Less frequent symptoms include vomiting, ascites, rectal bleeding, and jaundice. Studies have shown that EE can involve any segment of the intestinal tract [5]. Endoscopic findings are non-specific and may reveal erosions, erythema, and strictures. Additionally, abnormal laboratory findings include peripheral eosinophilia, elevated IgE levels, and specific food allergies [6]. In recent years, treatment has primarily focused on dietary modification, as many cases resolve spontaneously. The emergence of pharmacological modalities such as anti-histamines, leukotriene inhibitors, mast cell stabilizers, corticosteroids, and biologics has been helpful in treating refractory cases [7].

We present a unique case of a young female patient who presented to us at our emergency department (ED) with intractable vomiting, diarrhea, and severe dehydration. In the absence of fever and abdominal pain, she was managed as a suspected acute gastroenteritis. Her blood workup revealed peripheral eosinophilia, elevated serum IgE levels, and high C-reactive protein (CRP) and erythrocyte sedimentation rate (ESR) levels. Considering a wide range of potential pathologies, we came to the diagnosis of EE after excluding the secondary causes of peripheral eosinophilia and elevated IgE levels, and histopathological findings supporting the diagnosis. The secondary causes of peripheral eosinophilia such as parasitic and bacterial infections (e.g., *Helicobacter pylori*), inflammatory bowel disease, food allergies, neoplastic diseases (myeloproliferative disorders), celiac disease, immune-deficiencies (hyper-IgE syndrome), vasculitis (allergic vasculitis, eosinophilic granulomatosis with polyangiitis), and collagen vascular diseases (scleroderma) were excluded by the serological tests and imaging modalities.

As reported here, the intractable vomiting and diarrhea in the absence of other abdominal symptoms depicts the heterogeneity of EE and can pose diagnostic as well as therapeutic challenges for physicians.

## Case presentation

A 30-year-old female with a significant past medical history of asthma presented to our ED with a one-week history of intractable vomiting. At the onset of her illness, she experienced sudden, watery, large-volume, and odorless diarrhea. A day later, she developed vomiting that was sudden in onset, non-projectile, non-bilious, occurring four to five episodes per day, and not triggered by specific foods. Over time, her vomiting and diarrhea worsened to the extent of being unable to tolerate any type of food. On review of systems, she reported unintentional weight loss of approximately 12 pounds over the course of this week. There was no significant family history of atopy or known allergic disorders. She denied the use of tobacco, alcohol, and illicit drugs, and had no pet animals at home.

Her vitals upon admission were notable for a blood pressure of 80/60 mmHg, heart rate of 138 beats per minute, temperature of 97°F, respiratory rate of 18 breaths per minute, SaO_2_ of 95% on room air, and finger stick blood sugar of 93 mg/dL. On general physical examination, she had positive signs for severe dehydration: lethargic mentation, deeply sunken eyes, dry oral mucous membranes, and capillary refill of >5 sec. Rest of the physical examination was unremarkable.

Prior to admission, this patient was evaluated by multiple health care providers in other hospitals, where she underwent blood workup, X-ray of the abdomen, and ultrasound of the abdomen. However, her results were unremarkable, and she was discharged from the hospital with a treatment regimen for suspected diagnosis of acute gastroenteritis secondary to a viral etiology. Over the course of one-week treatment, she took oral metoclopramide 10 mg thrice daily, esomeprazole 40 mg once daily, ciprofloxacin 500 mg twice daily, and metronidazole 400 mg thrice daily. Her symptoms continued persistently and worsened to the point of not being able to tolerate any type of diet. Consequently, she presented to our ED with ongoing complaints of persistent vomiting and diarrhea.

Upon arrival at the ED, this patient was initially stabilized hemodynamically with boluses of intravenous (IV) fluid, followed by the maintenance fluid at the rate of 100 milliliters per hour. She was also administered injectable ondansetron 8 mg every 8 hours and omeprazole 40 mg once daily. All her oral medications were discontinued, and she was shifted to the intensive care unit.

**Figure 1:**
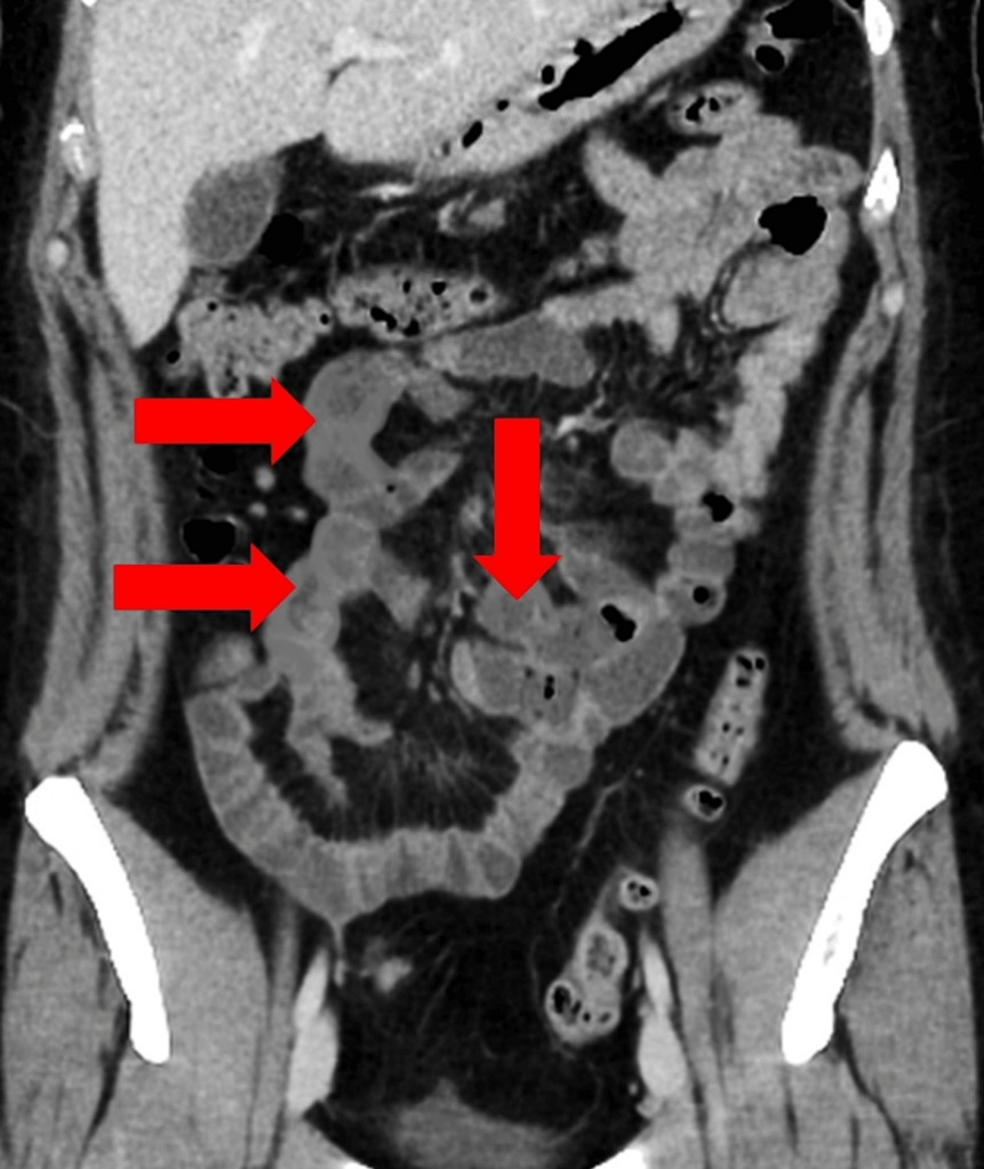
CT scan of the abdomen showing segmental thickening of the duodenum and jejunum gut walls.

On the day first of hospitalization, her symptoms didn’t improve significantly but she was vitally stabilized. Her initial blood workup was ordered on December 2, 2022, as shown in Table 1. Ultrasound of the abdomen and X-ray of the abdomen were normal. Computed tomography (CT) scan of the abdomen showed findings of multiple areas of segmental thickening of the bowel wall in the duodenum and proximal jejunum, as noted in Figure 1.

**Table 1:** Investigations over the course of hospitalization c-ANCA, cytoplasmic anti-neutrophil cytoplasmic antibody; CRP, C-reactive protein; dL, deciliter; ESR, erythrocyte sedimentation rate; fL, femtoliters; g, gram; HbsAg, hepatitis B surface antigen; IgE, immunoglobulin E; INR, international normalized ratio; L, liter; mg, milligram; mL, milliliter; p-ANCA, perinuclear, anti-neutrophil cytoplasmic antibody

Test	Reference Range	December 2, 2022	December 3, 2022	December 11, 2022
Hemoglobin (g/dL)	13.5–16.5	17	13	13.6
Mean corpuscular volume (fL)	76–96	77	76	74
Hematocrit	38.0	51	43	44
Platelet count (x10^9^/L)	150–400	756	437	215
White blood cells (x10^9^/L)	4–10	11	11.6	9.3
Neutrophils (%)	40–70	60	53	60
Eosinophils (%)	1–6	7	24	5
Absolute eosinophil count (x10^9^/L)		770	2784	465
Sodium (meq/L)	136–149	128	136	133
Potassium (meq/L)	3.8–5.2	2.7	3.3	4.1
Chloride (meq/L)	98–107	110	105	109
Blood urea nitrogen (mg/dL)	15–45	80	56	13
Calcium (meq/L)	8.5–10.2			
Creatinine (mg/dL)	0.6–1.3	1.8	1.2	0.9
Prothrombin time (seconds)	11–13.5	13		
INR		1.0		
Bilirubin (mg/dL)	0.1–1.2	0.6	0.8	
Gamma-glutamyl transferase (U/L)	0–25	14	13	
Alanine transaminase (U/L)	10–40	39	31	
Alkaline phosphatase (U/L)	24–147	128	130	
Aspartate transaminase (U/L)	10–34	40	23	
Albumin (g/dL)	2.4–4	4.0	4.5	
HBsAg		Negative		
Anti-hepatitis C antibody		Non-reactive		
Serum IgE	1.5–150		1411.8	413
Antinuclear antibody			Negative	
p-ANCA			Negative	
c-ANCA			Negative	
CRP	<6	70	30	5
ESR	0–20	140	134	60
Stool for *Helicobacter pylori *antigen			Negative	
Fecal calprotectin (μg/mg)	50–200			157
Stool analysis for ova, cyst, parasites, reducing sugars, and fat globules		Negative		
Anti-tissue transglutaminase–IgA			Negative	
Skin prick test	Negative			

**Figure 2:**
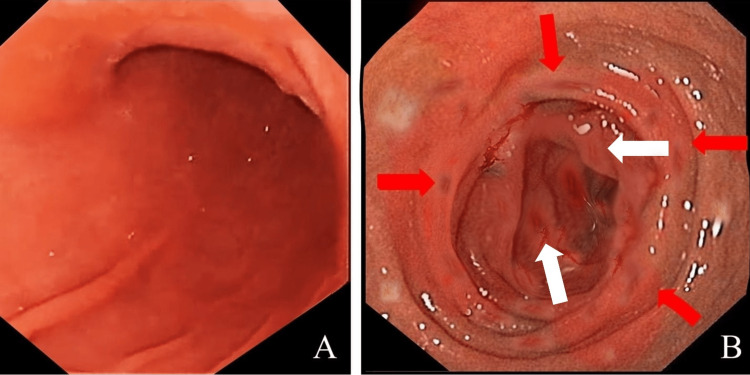
Endoscopy showing (A) normal gastric mucosa (B) patchy areas of gut wall thickening (white arrows), erythema, and erosions in the small bowel (red arrows).

Suspected for an underlying gastric outlet obstruction and/or inflammatory bowel disease, upper GI endoscopy was performed on the third day of hospitalization. Although non-specific, endoscopic findings ruled out gastric outlet obstruction but displayed patchy areas of erythema in the duodenum and proximal jejunum, as shown in Figure 2. The biopsy was taken from multiple segments of the intestine, and histopathology was ordered. With findings consistent with eosinophilic GI disease, i.e., peripheral eosinophilia, elevated IgE levels, and non-specific erosions on endoscopy, she was empirically started on dexamethasone infusion of 30 mg per day.

On the fifth day of hospitalization, her symptoms started improving. Her vomiting and diarrhea resolved completely by the tenth day in hospital. She was shifted to oral prednisolone 20 mg twice daily, montelukast 10 mg once daily, and ketotifen 2 mg once daily. A repeated blood count performed on December 11, 2022, showed normal eosinophil count, normal CRP levels, decreasing IgE levels, and declining pattern in ESR. Subsequently, she was discharged to home on oral medications.

**Figure 3:**
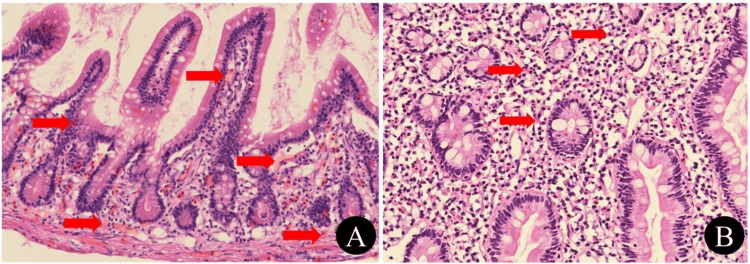
Histologic findings on endoscopic biopsy specimens using hematoxylin-eosin coloration. (A) Numerous eosinophilic degranulations are seen in the lamina propria and muscularis mucosae (red arrows). (B) Numerous eosinophils are also seen (>20 eosinophils per high-power field) (red arrows).

Upon follow-up a week later, this patient had no GI complaint, and a tapering regimen of prednisolone was planned at 5 mg per week for four weeks until discontinuation. The allergy food panel was found to be normal. Furthermore, her histopathology report, which was also available at the follow-up visit, revealed dense infiltrates of eosinophils with degranulation in the lamina propria and muscularis mucosa of the duodenum and jejunum, as shown in Figure 3. This confirmed our suspected diagnosis of EE.

## Discussion

Eosinophilic GI diseases are a rare group of chronic and immune-mediated conditions that can affect any segments of the GI tract, including esophagus (eosinophilic esophagitis), stomach (eosinophilic gastritis), small bowel (EE), and colon (eosinophilic colitis) [1]. Although eosinophilic esophagitis has garnered the most focus, non-esophageal enteritis manifestations such as eosinophilic gastritis, enteritis, and colitis are becoming more frequently encountered. The estimated prevalence of non-esophageal enteritis is 3-8/100,000 or ~50,000 cases in the United States, as suggested by the administrative databases [8]. A population-based study conducted in the United States between 2012 and 2017 estimated the prevalence of eosinophilic gastroenteritis and colitis to be 5.1/100,000 and 2.1/100,000 cases, respectively [1].

The term “eosinophilic enteritis” was first introduced by Kaijser in 1937 and has often been used interchangeably with eosinophilic gastroenteritis in literature, as the nomenclature is under review. It is defined by the presence of GI symptoms associated with eosinophil-rich infiltration of the intestinal mucosa, without secondary intestinal eosinophilia [2]. The exact epidemiology of EE is unknown, as there have been less than 400 cases reported in the literature. Similarly, its prevalence stands at approximately 1 case per 100,000 population. It can affect both genders, with a slightly higher predominance among females, as observed in our case. Additionally, it can manifest across all age groups as evidenced by various case reports and series [4].

The exact pathogenesis of this disorder remains elusive. While believed to have an allergic origin, this hypothesis is still debated due to the fact that only 70% of cases may exhibit a personal or family history of atopy and/or allergy [9]. In this patient, the high absolute eosinophil count and elevated serum IgE levels favor an allergic origin of EE. It is thought that numerous factors such as parasitic infections, bacterial infections, and allergens contribute to the activation of eosinophils in the GI wall. Activated eosinophils release acidotic cationic proteins (major basic protein, eosinophil cationic protein, eosinophil peroxidase, and eosinophil-derived neurotoxin) and pro-inflammatory cytokines (interleukin-4, interleukin-5, interleukin-13, and RANTES) [10]. These substances collectively disrupt cell membranes and activate adaptive immune cells (e.g., Th2 cells) within the gut wall, amplifying inflammation. Additional factors such as interleukin-33, eosinophilic-like receptors (C-C motif chemokine receptor 3), and IgE-mediated responses [11, 12] have also been proposed in the pathogenesis. Our patient had a personal history of asthma, but no significant family history of atopy and/or allergic disorder. Furthermore, her negative food allergen panel and skin prick test indicated that EE was likely triggered by elevated IgE levels and a personal history of atopy, even in the absence of an identifiable allergen.

In 1970, Klein et al. described three different forms of EE based on the intestinal layer involved: the mucosal, muscular, and subserosal. Of note, the clinical presentation can vary depending on the degree and depth of GI tract involvement. The mucosal form, often involving duodenum, is the most common variant. It typically presents with overlapping symptoms such as abdominal pain, vomiting, diarrhea, blood in stools, iron-deficiency anemia, malabsorption, protein-losing enteropathy, and failure to thrive. The muscularis form is characterized by thickening of the bowel wall, leading to symptoms related to GI obstruction. The serosal form, characterized by the exudative ascites, is rare [13]. Our patient had both mucosal and muscularis involvement, as evidenced by her symptoms, imaging, and histopathology. In some cases, patients have presented with an acute abdomen as their primary GI symptom [14]. We strongly believe that the initial presentation of diarrhea and vomiting in this patient represented an acute flare of EE. Moreover, the natural history of EE can be more complex, with acute flares that may lead to life-threatening hemodynamic instability, as seen in this patient.

The diagnosis of EE relies on a detailed history, thorough physical examination, and diagnostic investigations. The main abnormal finding in blood is an elevated eosinophilic count (eosinophils > 500/mm^3^), seen in nearly 20%-80% of patients [15]. Similarly, other non-specific laboratory findings such as elevated IgE levels, hypoalbuminemia, iron deficiency, and increased inflammatory markers such as CRP and ESR may also be present [16]. Imaging studies are highly non-specific and may show gut wall thickening. It may also help in excluding other secondary causes of GI disease [17]. Likewise, upper GI endoscopy may also show non-specific findings of erythema, erosions, edema, or nodules [18]. In our patient’s case, peripheral eosinophilia, elevated IgE levels, and high CRP and ESR levels were observed, with the latter two returning to baseline after treatment. In the absence of secondary causes of eosinophilia, the biopsy confirmed our final diagnosis of EE.

The treatment of EE poses a challenge due to the absence of clear guidelines. Non-pharmacological approaches involve the elimination of diets that trigger atopy and/or allergic reactions, although data on dietary elimination are insufficient in the current literature. Pharmacological therapies are the mainstay of treatment. Prednisolone is a widely used regimen, given at the dose of 1 mg per kg per day for few weeks followed by a gradual tapering over 6 to 8 weeks. Other therapies options include leukotriene inhibitors (e.g., montelukast 10 mg once daily), mast cell stabilizers (e.g., sodium cromoglycate 200 mg thrice daily), anti-histamines (e.g., ketotifen 1-2 mg twice daily), and medications such as azathioprine and biologics (e.g., mepolizumab, omalizumab, infliximab, adalimumab) [19, 20]. Our patient received IV dexamethasone initially and subsequently took oral prednisolone for a duration of eight weeks, along with montelukast and ketotifen for 12 weeks. She responded very well to this combination therapy. Moreover, during the three-month follow-up, no disease flare was observed.

## Conclusions

EE is a rare disorder characterized by a constellation of non-specific GI symptoms, laboratory findings, and imaging abnormalities. Our case presents a unique manifestation of EE, marked by intractable vomiting and diarrhea upon presentation, imitating the symptoms of acute gastroenteritis. In a case such as ours, it is essential to remember that EE can exhibit an acute flare and create diagnostic and therapeutic challenges for physicians. A high index of suspicion becomes imperative in such cases. Given the scarcity of literature on the acute flare aspect of EE, we emphasize the need for further extensive research to comprehend the diverse facets of this intricate disorder.
